# Distribution of device-measured 24-h movement behaviors in older adults: cross-sectional findings from the HUNT4 study

**DOI:** 10.1038/s41598-026-36355-y

**Published:** 2026-01-15

**Authors:** Karen Sverdrup, Astrid Ustad, Gro Gujord Tangen, Atle Kongsvold, Beatrix Vereijken, Bjørn Heine Strand, Geir Selbæk, Linda Ernstsen, Paul Jarle Mork

**Affiliations:** 1 Norwegian Centre for Ageing and Health, Vestfold Hospital Trust, Tønsberg, Norway; 2 Department of Geriatric Medicine, Oslo University Hospital, Oslo, Norway; 3 Department of Anesthesiology and Pain Medicine, University of Washington, Seattle, USA; 4 Department of Neuromedicine and Movement Science, Norwegian University of Science and Technology, Trondheim, Norway; 5 Department of Public Health and Nursing, Norwegian University of Science and Technology, Trondheim, Norway; 6 Department of Physical Health and Ageing, Norwegian Institute of Public Health, Oslo, Norway; 7 Faculty of Medicine, University of Oslo, Oslo, Norway; 8 Clinic of Medicine, St. Olavs Hospital, Trondheim University Hospital, Trondheim, Norway

**Keywords:** Epidemiology, Epidemiology

## Abstract

Comprehensive mapping of key physical activity (PA) types, postures, and sleep among older adults is important for informing public health policies and interventions. This study aimed to describe the 24-h time distribution of key PA types, postures, and sleep in a population-based sample of community-dwelling older adults and explore whether age, sex, and educational level influenced this distribution. Participants 65 years and older from the fourth survey of the Trøndelag Health Study (HUNT4, 2017–19) with ≥ 1 day of complete accelerometer recording were included (n = 8,114). PA types (walking, running, cycling), postures (standing, sitting, lying (awake)), and sleep were derived from the accelerometer data using validated machine learning models. Survey-weighted regression models were applied to describe the 24-h time distribution of PA types, postures, and sleep by age, sex, and education. Participants spent 4.1 h standing (*SD* 85.3 min), 82.8 min (*SD* 40.3 min) walking, 0.2 min (*SD* 1.7 min) running, 4.6 min (*SD* 7.2 min) cycling, 9.2 h (*SD* 115.5 min) sitting, 2.1 h (*SD* 86.6 min) lying (awake), and 7.1 h (*SD* 50.4 min) sleeping per day. Time spent standing and walking decreased, while time spent sitting, lying (awake), and sleeping increased with higher age. Women spent more time standing and sleeping, and less time walking, sitting, and lying (awake) than men. Higher education was associated with more time standing and walking and less time sitting. This study provides novel insights into the distribution of 24-h movement behaviors among older adults and can serve as a benchmark for future research on key PA types, postures, sleep, and their interactions.

**Supplementary Information:**

The online version contains supplementary material available at 10.1038/s41598-026-36355-y.

## Introduction

Evidence strongly supports the importance of physical activity (PA) for maintaining physical function and independence in older adults^1^. Conversely, excessive sedentary behavior increases the risk of numerous adverse health outcomes^2^. From age 60 onward, time spent in PA decreases while sedentary behavior increases, presenting a major public health challenge given the global ageing population^3^. Although historically studied separately, PA and sedentary behavior are interdependent movement behaviors that, together with sleep, make up the 24-h cycle^4^. Understanding the distribution and determinants of these behaviors has been identified as a key public health research priority^5^.

Traditionally, movement behaviors are typically measured using self-reports, which are prone to measurement errors and misclassification, particularly among older adults^6,7^. Epidemiological studies increasingly use accelerometry to overcome these limitations^8,9^. Typically, accelerometer data are analyzed by converting the acceleration signal into vector magnitude or counts within predefined time windows^10,11^, and by using age-specific activity thresholds (i.e., cut-points) to classify PA into intensity levels (e.g., light, moderate, vigorous PA). However, only about 15% of studies on PA in older adults use accelerometry^12^, and there is no consensus on activity intensity thresholds for this group^13–15^, which complicates comparisons across studies and hampers progress in the field. An alternative to classifying accelerometer data by PA intensity is to categorize data into key PA types (e.g., walking, running) and postures (e.g., standing, sitting, lying)^16–18^. Identifying key PA types and postures is important because they have different health effects^19,20^. Standing and walking are essential for independence and daily life participation, forming the main components of everyday PA, especially among older adults^21^. Moreover, sitting versus lying may reflect different levels of participation and functional capacity in older adults. Additionally, sleep occupies a significant portion of the 24-h cycle and is closely linked to daytime PA^22,23^.

Higher socioeconomic status is correlated with more PA^24,25^, and when comparing moderate-to-vigorous PA levels, men tend to be more active than women^3^. Some studies suggest that this sex difference persists throughout life^3^, while others indicate that it diminishes with age^26^. When considering light PA or specific PA types and postures, such as standing and walking, women appear more active than men^27^. Thus, knowledge of sex differences remains uncertain, particularly with increasing age. Currently, there is limited population-based data on the time distribution of 24-h movement behaviors and their determinants among older adults. Understanding this can inform interventions and public health policies to promote beneficial movement behaviors among older adults. Thus, this study aimed to describe the 24-h time distribution of key PA types, postures, and sleep in a population-based sample of community-dwelling older adults and explore whether age, sex, and educational level influenced this distribution.

## Methods

### Study population

This cross-sectional, population-based study used data from the fourth Trøndelag Health Study (HUNT4)^28^. Trained health professionals conducted interviews and clinical assessments at a test station. For participants ≥ 70 years unable to meet at the test station, interviews and clinical assessments were offered at their primary residence, whether at home or in a nursing home. Data collection took place between August 2017 and February 2019.

All participants, or their closest proxies, provided written informed consent before participation. Participant’s ability to consent was based on the judgement of the assessors and the health personnel in the nursing homes. Ethical approval was granted by the Regional Committee for Ethics in Medical Research, South-East B (ref. 461,091). The Norwegian Agency for Shared Services in Education and Research conducted and approved the Data Protection Impact Assessment (ref. 233754). All methods were carried out in accordance with relevant guidelines and regulations.

**Fig. 1:**
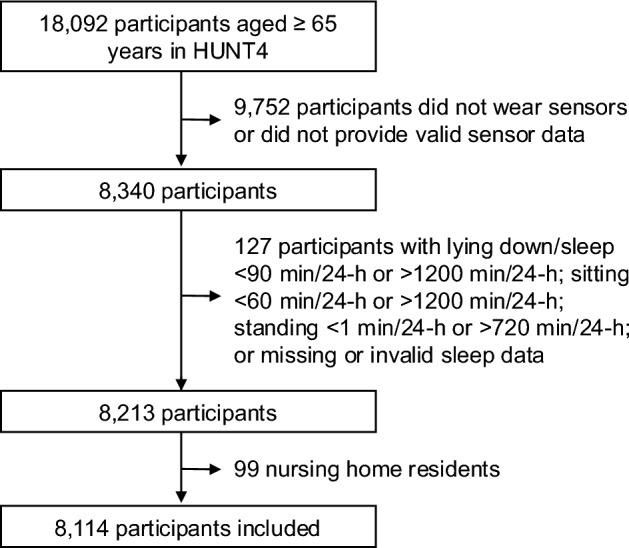
Participant inclusion flowchart. Note: HUNT4 = the Trøndelag Health Study, 4th survey.

All participants ≥ 65 years in HUNT4 (N = 18,092) were eligible for inclusion. Figure 1 shows the participant inclusion flowchart. In total, 8,340 of the eligible participants provided dual-accelerometer data (thigh and low back) for ≥ 1 day (midnight to midnight). Participants were excluded if they had exceptionally short or long time in lying / sleep (< 90 min/24-h or > 1,200 min/24-h), sitting (< 60 min/24-h or > 1,200 min/24-h) and standing (< 1 min/24-h or > 720 min/24-h) (n = 85), or had missing or invalid sleep data (n = 42). In addition, nursing home residents (*n* = 99) were excluded from the analytical sample as specific environmental factors influence their everyday lives^29^. In total, 8,114 participants were included in this study. Participants included in the study were younger, had higher education, were more often married, and had better self-rated health than HUNT4 participants aged ≥ 65 years who were not included (all *p* ≤ 0.05, Table S1).

### 24-h movement behaviors

At the end of the clinical examination in HUNT4, participants were asked to wear two AX3 accelerometers (Axivity, Newcastle, UK) for seven days. Detailed information about the accelerometer data collection procedures has been described elsewhere^7^. In brief, one accelerometer was placed on the right thigh and the other on the lower back. The accelerometer data was sampled at 50 Hz with 8G bandwidth and stored offline on the AX3 internal flash drive. After completion, the participants either returned the accelerometers in a pre-stamped envelope or delivered them back at the examination site.

The OmGui software (v 1.0.0.43; Open movement, Newcastle, UK) was used to configure, initialize, and download the data. The data streams from the two accelerometers were synchronized and segmented into 5-s windows. A validated machine learning (ML) model was used to classify key PA types and postures, including standing, walking, running, cycling, sitting, and lying (awake)^17,30^. A separate ML model that incorporated data from the temperature sensor embedded in the AX3, was used to classify non-wear time^31^. If non-wear was predicted for at least one hour, the entire 24-h period was excluded from further analysis. Furthermore, the first and last measurement days were excluded (i.e., attaching and removing accelerometers). Finally, a separate ML model based on accelerometer data, temperature recordings, and sleep patterns validated against polysomnographic recording was used to predict sleep duration^32^. These ML models have been shown to achieve high overall accuracy in predicting PA types and postures (96%)^17^, non-wear time (97%)^31^, and sleep duration (84%)^32^. The time spent in each movement behavior was averaged across valid days and reported as average minutes per 24-h (min/24-h) in all tables and figures. In the text, movement behaviors with durations ≥ 90 min/24-h are reported as hours/24-h.

### Background characteristics

Age, sex, education, and marital status at participation were obtained from Statistics Norway and linked to each participant in the study sample using the unique personal identification numbers allocated to all Norwegian residents. Education was recorded according to the International Standard Classification of Education 2011 as the highest level achieved: Primary (≤ 10 years), Secondary (11–13 years), and Higher (≥ 14 years). For regression analyses, education was dichotomized into “Primary/Secondary” (< 14 years) and “Higher” (≥ 14 years). At the year of participation, marital status was registered as “Married,” “Unmarried,” “Divorced,” or “Widow/Widower.” Weight and height were measured with participants dressed in light clothing, without shoes, using a digital flat scale (Seca 813, Germany) and a stadiometer (Seca 217, Germany) during the clinical examination. Body mass index (BMI) was calculated by dividing weight (kg) by the square of height (m^2^) and categorised as underweight (< 18.5 kg/m^2^), normal weight (18.5–24.9 kg/m^2^), overweight (25–29.9 kg/m^2^), and obesity (≥ 30 kg/m^2^). Self-rated health was obtained from a questionnaire completed before the clinical examination and assessed using the question “How is your health at the moment?”^33^, with response options “Poor,” “Not so good,” “Good,” and “Very good.”

### Statistical analysis

All analyses were performed using Stata 18. To address potential selection bias for participants not included in the analytical sample, inverse probability weighting (IPW) was applied. The IPW was based on demographic variables (age, sex, education, and marital status) and self-rated health. IPW procedures are provided in Table S2 and Fig. S1. Crude estimates are provided in Tables S3 and S4.

Background characteristics and 24-h movement behaviors were described as weighted means (*SD*) for continuous variables and unweighted counts (n) with weighted percentages for categorical variables. Survey-weighted regression models were used to analyze standing, walking, sitting, lying (awake), and sleeping by sex across age. Age was modelled using linear and quadratic terms and cubic splines (3 and 4 knots) to account for potential non-linear relationships between age and movement behavior. Model fit was evaluated using likelihood-ratio test (LRT), Akaike’s Information Criterion (AIC), and Bayesian Information Criterion (BIC). The model that best balanced fit and complexity was selected. The choice of model varied by behavior: cubic splines (3 knots) were selected for sitting, standing, and walking, whilst quadratic models were selected for sleeping and lying (awake). Interaction terms between age and sex were included to assess differences in these relationships by sex. Marginal means with 95% confidence intervals (CI) were plotted to visualize movement behaviors by sex across age. To examine the effect of sex with age, conditional marginal effects of sex were plotted.

Survey-weighted regression models were used to analyze standing, walking, sitting, lying (awake), and sleeping by education and sex across age. Also, in these analyses, age was modelled using linear and quadratic terms and cubic splines (3 knots), with model selection guided by LRT, AIC, and BIC. Quadratic models were selected for standing, walking, sitting, lying, and sleeping for both sexes. Interaction terms between age and education were included to assess differences in these relationships by education. Marginal means with 95% CIs and conditional marginal effects of sex were plotted to visualize movement behavior by education for women and men across ages. Conditional marginal effects were plotted to examine the effect of education by sex with age.

## Results

### Background characteristics

Table 1 shows the background characteristics of all study participants. In total, 52.6% were women, and the mean age was 74.7 (*SD* 7.2) years ranging from 65 to 100 years. In total, 24%, 54.8%, and 21.2% had primary, secondary, and higher education, 63.3% were married, and 65.1% reported good/very good self-rated health. In higher age groups, fewer had higher education, more were widowed or widowers, and fewer reported good/very good self-rated health (Table S5).

**Table 1:** Weighted background characteristics for all study participants.

	Total	Women	Men
	(N = 8,114)	(n = 4,266)	(n = 3,848)
Age, years (range 65–100), mean (*SD*)	74.7 (7.2)	75.1 (7.5)	74.2 (6.9)
Education, years, n (%)			
≤ 10	1,519 (24.0)	920 (27.6)	599 (19.9)
11–13	4,486 (54.8)	2,276 (52.3)	2,210 (57.7)
≥ 14	2,109 (21.2)	1,070 (20.1)	1,039 (22.4)
Marital status, n (%)			
Unmarried	382 (5.3)	160 (4.1)	222 (6.7)
Divorced	948 (11.2)	544 (11.9)	404 (10.4)
Widow/ -er	1,313 (20.2)	1,014 (29.7)	299 (9.4)
Married	5,471 (63.3)	2,548 (54.3)	2,923 (73.4)
Self-rated health, n (%)			
Poor	96 (2.5)	50 (2.6)	46 (2.5)
Not so good	2,139 (32.4)	1,207 (34.8)	932 (29.7)
Good	4,895 (57.6)	2,486 (55.4)	2,409 (60.0)
Very good	754 (7.5)	385 (7.2)	369 (7.8)
BMI, kg/m^2^, mean (*SD*)	27.2 (4.2)	26.9 (4.6)	27.5 (3.8)
Underweight, n (%)	51 (0.7)	49 (1.2)	2 (0.1)
Normal weight, n (%)	2,387 (28.2)	1,443 (31.9)	944 (24.0)
Overweight, n (%)	3,704 (44.3)	1,707 (38.5)	1,997 (50.9)
Obesity, n (%)	1,928 (26.8)	1,051 (28.3)	877 (25.1)

Note: Continuous variables are shown as weighted means (*SD*), and categorical variables as unweighted counts (n) with weighted percentages (%). BMI = body mass index; kg = kilograms; m = meters.

### 24-h movement behaviors

Table 2 shows the distribution of movement behaviors for the total study sample and by sex. Over 97% of women and men provided ≥ 3 days of accelerometer data. On average, women spent 4.4 h (*SD* 85.7 min) standing, 80.2 min walking (*SD* 39.4 min), 0.1 min running (*SD* 0.7 min), 3.5 min cycling (*SD* 5.4 min), 9.0 h sitting (*SD* 111.8 min), 2.0 h lying (awake) (*SD* 80.5 min), and 7.2 h sleeping (*SD* 49.1 min) per day. Men spent 3.8 h (*SD* 80.9 min) standing, 85.7 min walking (*SD* 41.1 min), 0.4 min running (*SD* 2.3 min), 5.7 min cycling (*SD* 8.7 min), 9.4 h sitting (*SD* 118.5 min), 2.3 h lying (awake) (*SD* 92.1 min), and 7.0 h sleeping (*SD* 50.9 min) per day.

**Table 2:** Weighted movement behavior (min/24-h) for all study participants (age ranged from 65–100 years).

	Total	Women	Men
	(N = 8,114)	(n = 4,266)	(n = 3,848)
Wear time accelerometers			
Days, mean (*SD*)	5.8 (0.9)	5.7 (0.9)	5.8 (0.9)
1–2 days, n (%)	193 (2.6)	114 (2.9)	79 (2.2)
≥ 3 days, n (%)	7 921 (97.4)	4 152 (97.1)	3 769 (97.8)
Movement behavior, mean (*SD*)			
Standing	247.8 (85.3)	264.3 (85.7)	229.1 (80.9)
Walking	82.8 (40.3)	80.2 (39.4)	85.7 (41.1)
Running	0.2 (1.7)	0.1 (0.7)	0.4 (2.3)
Cycling	4.6 (7.2)	3.5 (5.4)	5.7 (8.7)
Sitting	551.2 (115.5)	540.7 (111.8)	563.1 (118.5)
Lying (awake)	127.8 (86.6)	119.4 (80.5)	137.2 (92.1)
Sleeping	424.9 (50.4)	431.0 (49.1)	418.0 (50.9)

Note: Continuous variables are shown as weighted means (*SD*), and categorical variables as unweighted counts (*n*) with weighted percentages (%).

**Fig. 2:**
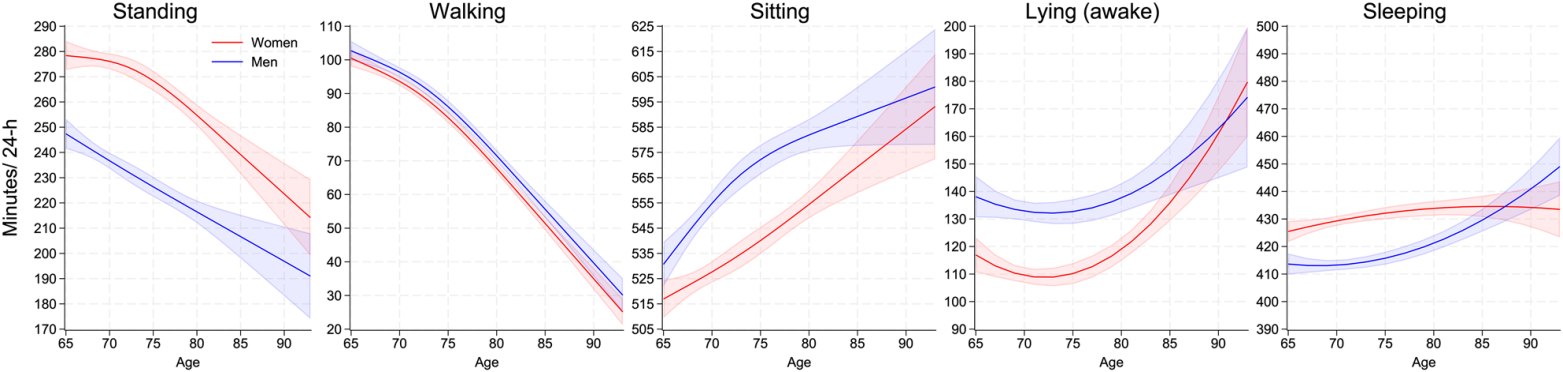
Movement behavior (min/24-h) by sex across age. Legends: Predicted average daily minutes standing, sitting, lying (awake), and sleeping, with 95% confidence intervals by sex across age. Red: women. Blue: men. The y-axis range varies to reflect the range of each behavior.

Figure 2 shows the estimated distribution of time spent standing, walking, sitting, lying (awake), and sleeping for women and men across age. With higher age, women and men spent less time standing and walking and more time sitting, lying (awake), and sleeping. Movement behavior (min/ 24-h) by age group and sex is reported in Table S6. Women spent significantly more time standing and sleeping (across age and until ~ 85 years, respectively) and less time walking (between ~ 70–85 years), sitting (until ~ 85 years), and lying (awake) (until ~ 85 years) than men (Fig. S2).

**Fig. 3:**
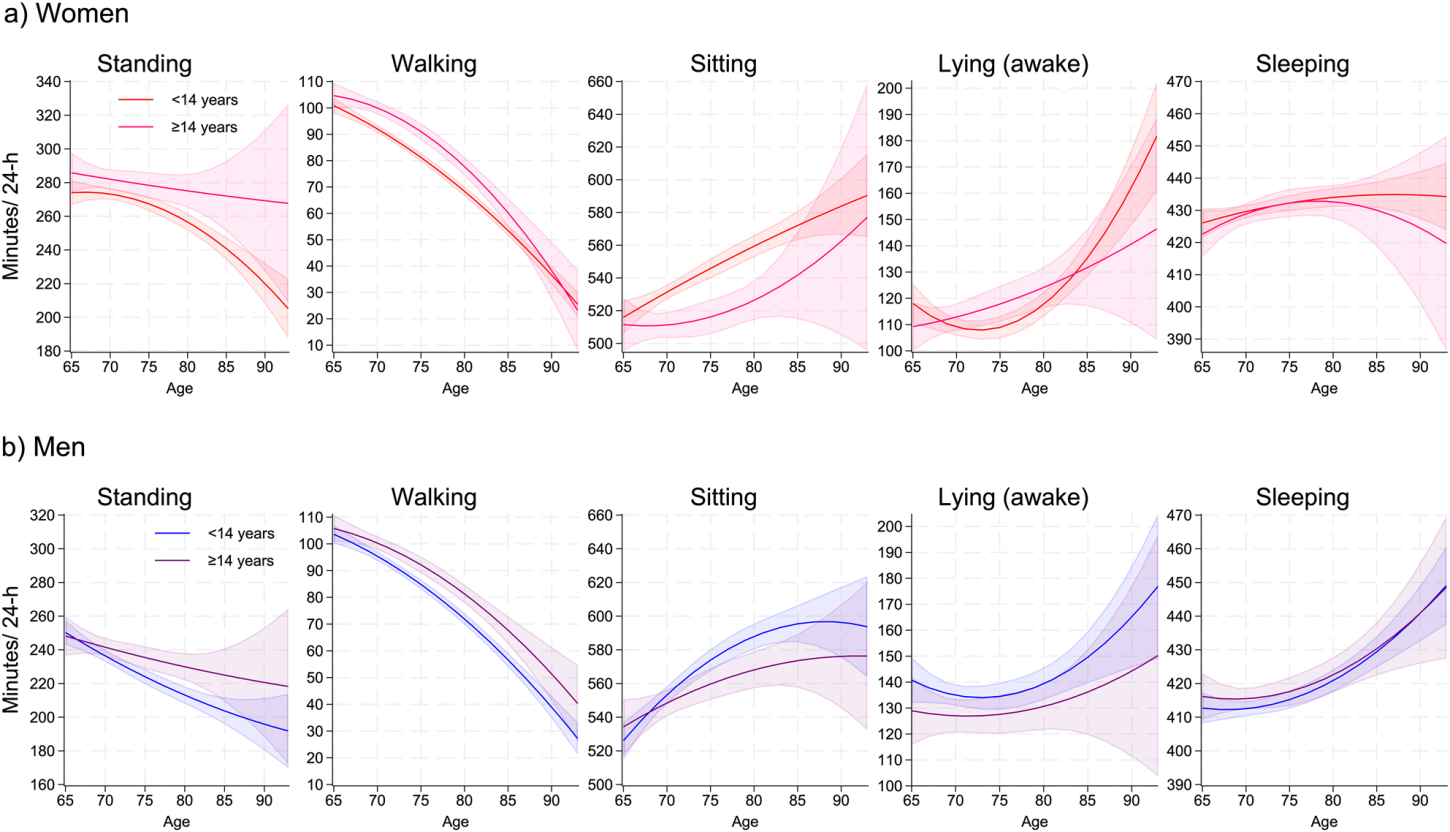
Movement behavior (min/24-h) by sex and level of education across age. Legends: a) Predicted average daily minutes standing, sitting, lying (awake), and sleeping, with 95% confidence intervals for women by education across age. Red: < 14 years education. Pink: ≥ 14 years education. b) Predicted average daily minutes standing, sitting, lying (awake), and sleeping, with 95% confidence intervals for men by education across age. Blue: < 14 years education. Purple: ≥ 14 years education. The y-axis range varies to reflect the range of each behavior.

Figure 3 shows the estimated distribution of time spent standing, walking, sitting, lying (awake), and sleeping for women and men with primary/secondary (< 14 years) and higher (≥ 14 years) education across age. For both sexes, older adults with primary/secondary education spent significantly less time standing (until ~ 90 years for women and between ~ 70–85 years for men) and walking (between ~ 65–85 for women and ~ 70–90 years for men) and more time sitting (between ~ 65–85 years for women and ~ 75–85 years for men) than women and men with higher education (Fig. S3).

## Discussion

This population-based cross-sectional study explored the 24-h distribution of movement behaviors by age, sex, and educational level in one of the largest existing cohorts of older adults (≥ 65 years). On average, older adults spent most of the day sitting, followed by sleeping, standing, lying (awake), and walking. With higher age, time spent standing and walking decreased, while time spent sitting, lying (awake), and sleeping increased. Women spent more time standing and sleeping and less time walking, sitting, and lying (awake) compared to men. Both women and men with higher education spent more time standing and walking and less time sitting than those with primary or secondary education.

Older adults in this population-based cohort spent considerably more time standing (4.1 h/day) compared to adults in a recent pooled study cohort (3.2 h/day, N = 14,761, average age 54 years)^34^, as well as older adults in the Copenhagen City Heart Study (3.0 h/day, N = 1,670, average age 72.7 years)^35^. In comparison, the oldest age group in our study (≥ 90 years) spent a similar amount of time standing as the average in these two other studies^34,35^. This supports the idea that standing is the primary awake movement behavior^21^ and a key contributor to total volume of PA in older adults^28^. Across age, older women spent more time standing than men, which challenges the general view that women are less physically active than men^3,26^, and supports that women stand more^35^ and engage in larger volumes of light PA than men^27^. Further, women and men with higher education spent more time standing across age groups than those with primary or secondary education, expanding current knowledge on socioeconomic differences in PA^24,25^ to include standing behavior. Recent technological advances have allowed the identification of standing as a separate behavior, and more time spent standing has been associated with lower mortality risk and better cardiometabolic health^20,36^. Standing has been proposed as an accessible activity to help counteract prolonged sedentary behavior^37^. Conversely, from a 24-h movement perspective, replacing higher-intensity PA with time standing has been associated with worse cardiometabolic outcomes^34^. This emphasizes the interconnectedness of these behaviors and highlights the importance of considering the full 24-h movement profile when assessing a behavior’s health effects. Since older adults spend a significant amount of time standing, which can now be distinguished from other forms of light PA, the independent and compositional effects of standing on health outcomes in older adults warrant further investigation.

Older adults in this study spent the same amount of time walking per day (82.8 min) as those in the Copenhagen City Heart Study (82.6 min/day)^35^. However, they spent considerably less than the average reported in the pooled study cohort (2.7 h/day)^34^, and significantly more than reported in the United States National Health and Nutrition Examination Survey (28 min/day, N = 3,725, average age not reported)^38^. In addition to differences in age, education levels, and self-reported health, variations in accelerometer setups and movement behavior classification models complicate direct comparisons across studies, making conclusions uncertain and potentially misleading. In our study, time spent walking decreased with higher age for both women and men, consistent with previous findings^39^. However, up to age 80 for women and 85 for men, their walking time exceeded the approximately 64 min per day previously associated with a favorable cardiometabolic risk profile^19^. Walking has been associated with numerous health benefits, including reduced risk of cardiovascular disease, dementia, and mortality^34,39–41^. Since older adults have lower resting metabolic rates and higher energy costs for any given PA than younger adults^42^, this could potentially amplify the health benefits of walking in this population^41,43,44^. Exceeding the walking duration associated with favorable cardiometabolic risk profiles until reaching their eighties, combined with the relatively high proportion of study participants rating their health as good or very good (65.1%), suggests that the older adults in this study are relatively healthy and high functioning. We also observed that men walked more than women, although the difference was small, ranging from 2.5 to 5.0 min (in the oldest age group, the difference increased to 8.5 min). Additionally, women and men with higher education spent more time walking than those with primary or secondary education, indicating that socioeconomic disparities in PA persist into older age.

On average, older adults spent 0.2 min running and 3.5 min cycling each day. This is less time spent running than across cohorts within the pooled study cohort (0.2–0.4 min/day)^19^, but similar to the amount reported in the Copenhagen City Heart Study (0.1 min/day)^35^. Regarding cycling, older adults in the current study spent more time cycling than those in the Copenhagen City Heart Study (1.2 min/day)^35^. As with walking, demographic, clinical, and methodological differences across studies make direct comparisons difficult. Regardless, running and walking make up a very small proportion of the 24-h day and may be better examined over a weekly period.

Older adults spent an average of 9.2 h sitting and 2.1 h lying (awake) per day. Combined, these behaviors total 11.3 h per day sedentary (any awake activity with energy expenditure ≤ 1.5 metabolic equivalents), which is higher than what other studies have reported^21,34,35,38^. The negative effects of excessive sedentary behavior on health outcomes are well documented^25^, but no other studies have distinguished between sitting and lying as forms of sedentary behavior. Although energy expenditure in sitting and lying positions is similar^42^, these postures may have different health effects beyond energy expenditure. Lying can promote relaxation and increase blood flow to internal organs, muscles, and skin^45^, and help counteract blood pooling in leg veins caused by prolonged sitting. Conversely, sitting may be associated with higher levels of participation and function in older adults^46^. We further observed that time spent sitting and lying (awake) increased with age, that women spent less time sitting and lying (awake) than men, and that older adults with higher education spent significantly less time sitting than those with primary or secondary education. Thus, older women and men exhibit different daily movement patterns: women stand more often but walk, sit, and lie less than men do. This adds detail to sex differences in PA and sedentary behavior among older adults and supports continued sex-specific research on how different movement behaviors affect health in this group. Additionally, the finding that higher-educated individuals spend more time standing and walking than those with primary or secondary education highlights the previously documented importance of equal opportunity and access to education in promoting population-level PA^24^. Given that older adults spent most of their awake time sitting and lying, underscoring their vulnerability to adverse health outcomes, future research should explore how these behaviors influence health and PA initiatives targeting older adults that include strategies to address social disadvantages are essential to reduce this group’s vulnerability to adverse health outcomes.

Finally, a unique feature of this study is the differentiation of sleep from awake sedentary time. Our results are not affected by previous limitations of accelerometer-classified sleep, such as “non-movement” time within predefined hours of the day (e.g. 24.00–06.00)^47^. However, we did not measure consecutive sleep or the timing of sleep within the 24-h cycle. Still, older adults spent on average 7.1 h sleeping, which is in line with current sleep recommendations of 7–8 h per day^48^. Further, we found that, with higher age, sleep duration increased slightly for men but remained relatively stable for women. In comparison, previous research based on self-reported sleep and polysomnography has reported contrasting findings, with different studies showing increased, decreased, or stable sleep duration with higher age^49,50^. Further, our study showed that women sleep more than men, which aligns with previous research^49,50^. Sleep, however, encompasses more than duration. While women typically report more sleep problems than men, polysomnographic recordings indicate that women have better sleep quality^49,51^. Much remains to be understood about the interdependent relationships among sleep, PA types, and postures, and their impact on health in older adults.

This study has several strengths, including a large population of older adults and using validated ML models to classify key PA types, postures, and sleep with high overall accuracy. However, some limitations should be considered. First, we used data from a population-based cohort but excluded nursing home residents. Thus, results are not generalizable to that population group. Second, due to the availability of accelerometers and time constraints during the clinical examination, not all participants were asked to wear accelerometers. Information on “not asked”, “asked but declined”, and “asked and accepted” was unavailable in the data. Furthermore, among the participants who wore sensors, information on who did not provide accelerometer data (e.g., did not return the device, device recording failure) was not available. Consequently, we were only able to distinguish between participants who provided vs those who did not wear or did not provide sensor data. Participants who provided accelerometer data were younger, had higher education, and reported better self-rated health than those who did not (see Additional file 1). To account for this, analyses were weighted (see Additional file 2). Third, despite the high overall accuracy of the ML models, misclassifications can still occur, especially for standing versus walking^52^. Further, some participants’ data (n = 127) were excluded due to exceptionally short or long time in various postures and missing or invalid sleep data, which indicate misclassifications, undetected non-wear time, or accelerometer malfunction. However, this affected a small proportion of the study population (1.5%). Fourth, all movement behavior in this study is based on summarized time windows, and we lack information on other important aspects of these behaviors, such as timing, bout length, or the context in which they were performed. Lastly, our cross-sectional study design does not permit conclusions about longitudinal trajectories of 24-h movement behavior across age.

## Conclusions

Older adults in this large population-based cohort spent most of the 24-h cycle sitting, followed by sleeping, standing, lying (awake), and walking. From age 65 onward, daily standing and walking time declined, while sitting and lying increased. Women and men exhibit different daily movement patterns: women stand more but walk, sit, and lie less than men. Furthermore, older adults with higher education spent more time standing and walking and less time sitting than those with lower education. These findings highlight substantial age-related, sex, and socioeconomic differences in movement behaviour and underscore the need for policies and interventions that aim to reduce prolonged sitting and promote light PA among older adults. This population-based study can serve as a benchmark for future research on key PA types, postures, sleep, and their interactions in older adults.

## Supplementary Information

Supplementary Information.

## Data Availability

The data supporting the findings of this study are available from the HUNT Research Centre ([https://www.ntnu.edu/hunt)), but restrictions apply to these data, which were used under license for the current study. However, data are available from co-author “Paul Jarle Mork” (Email ID: [paul.mork@ntnu.no](mailto:paul.mork@ntnu.no)) upon reasonable request and with permission from the HUNT Research Centre ([https://www.ntnu.edu/hunt)).

## Abbreviations

PA: Physical activity
HUNT4: The Trøndelag Health Study, 4th survey
ML: Machine learning
BMI: Body mass index
IPW: Inverse probability weighting
LRT: Likelihood-ratio test
AIC: Aikake information criterion
BIC: Bayesion information criterion
CI: Confidence interval
