# Supplementary material for: Distribution of device-measured 24-h movement behaviors in older adults: cross-sectional findings from the HUNT4 study
Source: Sci Rep. 2026 Jan 15;16:5268. doi: 10.1038/s41598-026-36355-y (PMC12881634; doi:10.1038/s41598-026-36355-y)
Supplement: Supplementary file 1 — Supplementary Information. [file 41598_2026_36355_MOESM1_ESM.docx]

Device-measured 24-hour movement behavior in older adults. Cross-sectional findings from the HUNT4 study

Karen Sverdrup^1,2,3^, Astrid Ustad^4^, Gro Gujord Tangen^1,2^, Atle Kongsvold^5^, Beatrix Vereijken^4^, Bjørn Heine Strand^1,2,6^, Geir Selbæk^1,2,7^, Linda Ernstsen^5,8^, and Paul Jarle Mork^5,*^

1 Norwegian Centre for Ageing and Health, Vestfold Hospital Trust, Tønsberg, Norway

2 Department of Geriatric Medicine, Oslo University Hospital, Oslo, Norway

3 Department of Anesthesiology and Pain Medicine, University of Washington, Seattle, USA

4 Department of Neuromedicine and Movement Science, Norwegian University of Science and Technology, Trondheim, Norway

5 Department of Public Health and Nursing, Norwegian University of Science and Technology, Trondheim, Norway

6 Department of Physical Health and Ageing, Norwegian Institute of Public Health, Oslo, Norway

7 Faculty of Medicine, University of Oslo, Oslo, Norway

8 Clinic of Medicine, St. Olavs Hospital, Trondheim University Hospital, Trondheim, Norway

* [paul.mork@ntnu.no](mailto:paul.mork@ntnu.no)

**Supplementary Materials, content:**

**Supplementary Table S1**. Sensitivity analyses………………………………….……. p. 2

**Supplementary Table S2**. Logistic regression for inverse probability weight calculation……………………………………………………………………….….……. p. 3

**Supplementary Figure S1**. Distribution of inverse probability weights………..……. p. 3

**Supplementary Table S3**. Crude estimates of background characteristics and movement behavior (min/24-hour) for all study participants and by sex….…...….. p. 4

**Supplementary Table S4**. Crude estimates of background characteristics and movement behavior (min/24-hour) by sex and age group...…………………..……. p. 5

**Supplementary Table S5**. Weighted background characteristics by sex and age group………………………………………………………………………………..……. p. 6

**Supplementary Table S6**. Weighted movement behavior (min/24-hour) by sex and age group…………………………………………………………………….…….……. p. 7

**Supplementary Figure S2**. Predicted marginal effects of sex (men) across age on

24-hour movement behavior………………………………………………..……..…… p. 8

**Supplementary Figure S3**. Predicted marginal effects of education (<14 years) on

24-hour movement behavior……………………………………………...…..……...... p. 8

**Supplementary Table S1**. Sensitivity analyses

|  | **HUNT4 ≥65 years (N = 18,092)** | | | **Wore sensors (n = 8,340)** | | | **Valid wear (n = 8,213)** | | |
| --- | --- | --- | --- | --- | --- | --- | --- | --- | --- |
|  | **Did not wear sensors** | **Wore sensors** |  | **Invalid wear** | **Valid wear** |  | **Nursing home residents** | **Analytical sample** |  |
|  | **(n = 9,752)** | **(n = 8,340)** | **p** | **(n = 127)** | **(n = 8,213)** | **p** | **(n = 99)** | **(n = 8,114)** | **p** |
| Age, years, mean (SD) | 75.7 (7.8) | 73.7 (6.5) | <.001 | 75.7 (8.2) | 73.7 (6.5) | <.001 | 87.2 (7.3) | 73.5 (6.3) | <.001 |
| Age group, n (%) |  |  | <.001 |  |  | .002 |  |  | <.001 |
| 65–69 years | 2,770 (28.4) | 2,810 (33.7) |  | 34 (26.8) | 2,776 (33.8) |  | 0 (0) | 2,776 (34.2) |  |
| 70–74 years | 2,567 (26.3) | 2,554 (30.6) |  | 33 (26.0) | 2,521 (30.7) |  | 8 (8.1) | 2,513 (31.0) |  |
| 75–79 years | 1,710 (17.5) | 1,595 (19.1) |  | 29 (22.8) | 1,567 (19.1) |  | 10 (10.1) | 1,557 (19.2) |  |
| 80–84 years | 1,250 (12.8) | 814 (9.8) |  | 12 (9.4) | 802 (9.8) |  | 14 (14.1) | 788 (9.7) |  |
| 85–89 years | 885 (9.1) | 317 (4.4) |  | 10 (7.9) | 362 (4.4) |  | 26 (26.3) | 335 (4.1) |  |
| ≥90 | 570 (5.8) | 195 (2.3) |  | 9 (7.1) | 186 (2.3) |  | 41 (41.4) | 145 (1.8) |  |
| Sex, n (%) |  |  | .1 |  |  | .6 |  |  | .4 |
| Women | 5,254 (53.9) | 4,392 (52.7) |  | 70 (55.1) | 4,322 (52.6) |  | 56 (56.6) | 4,266 (52.6) |  |
| Men | 4,498 (46.1) | 3,948 (47.3) |  | 57 (44.9) | 3,891 (47.4) |  | 43 (43.4) | 3,848 (47.4) |  |
| Education, years, n (%) |  |  | <.001 |  |  | .08 |  |  | <.001 |
| 0–10 | 2,739 (28.1) | 1,595 (19.1) |  | 34 (26.8) | 1,561 (19.0) |  | 42 (42.4) | 1,519 (18.7) |  |
| 11–13 | 5,330 (54.7) | 4,593 (55.1) |  | 65 (51.2) | 4,528 (55.1) |  | 42 (42.4) | 4,486 (55.3) |  |
| ≥14 | 1,680 (17.2) | 2,152 (25.8) |  | 28 (22.0) | 2,124 (25.9) |  | 15 (15.2) | 2,109 (26.0) |  |
| Marital status, n (%) |  |  | <.001 |  |  | .01 |  |  | <.001 |
| Unmarried/ Divorced/ Widow/-er | 3,914 (40.1) | 2,770 (33.2) |  | 55 (43.3) | 2,715 (33.1) |  | 72 (72.7) | 2,643 (32.6) |  |
| Married | 5,838 (59.9) | 5,570 (66.8) |  | 72 (56.7) | 5,498 (66.9) |  | 27 (27.3) | 5,471 (67.4) |  |
| Self-rated health, n (%) |  |  | <.001 |  |  | <.001 |  |  | <.001 |
| Poor/ Not so good | 3,575 (39.6) | 2,327 (28.9) |  | 53 (46.5) | 2,274 (28.7) |  | 39 (76.5) | 2,235 (28.4) |  |
| Good/ Very good | 5,441 (60.4) | 5,722 (71.1) |  | 61 (53.5) | 5,661 (71.3) |  | 12 (23.5) | 5,649 (71.6) |  |
| Invalid wear was defined as exceptionally short or long time in lying/sleep (<90 min/day and >1200 min/day), sitting (<60 min/day and >1200 min/day), and standing (<1 min/day and >720 min/day) and missing or invalid sleep data  *p* ttest for normally distributed continuous variables, Chi^2^ for categorical variables. | | | | | | | | | |

**Supplementary Table S2**. Logistic regression for inverse probability weight calculation

| **Participation (ref. 0)** | **OR** | **SE** | **p** | **CI** |
| --- | --- | --- | --- | --- |
| Age group (ref. 65-69) |  |  |  |  |
| 70-74 | 1.03 | 0.04 | .4 | 0.96-1.11 |
| 75-79 | 1.03 | 0.05 | .5 | 0.94-1.12 |
| 80-84 | 0.79 | 0.04 | <.001 | 0.71-0.87 |
| 85-89 | 0.53 | 0.04 | <.001 | 0.46-0.61 |
| ≥90 | 0.40 | 0.04 | <.001 | 0.32-0.49 |
| Sex (ref. women) |  |  |  |  |
| Men | 0.95 | 0.03 | .08 | 0.89-1.01 |
| Education (ref. 0-10) |  |  |  |  |
| 11–13 | 1.27 | 0.05 | <.001 | 1.18-1.37 |
| ≥14 | 1.71 | 0.08 | <.001 | 1.56-1.88 |
| Marital status (ref. unmarried) |  |  |  |  |
| Divorced | 1.32 | 0.09 | <.001 | 1.15-1.51 |
| Widow/ -er | 1.17 | 0.09 | .04 | 1.01-1.37 |
| Married | 1.28 | 0.10 | .002 | 1.09-1.50 |
| Self-rated health (ref. poor) |  |  |  |  |
| Not so good | 1.89 | 0.23 | <.001 | 1.48-2.38 |
| Good | 2.58 | 0.31 | <.001 | 2.03-3.26 |
| Very good | 3.40 | 0.45 | <.001 | 2.62-4.41 |
| Missing | 1.12 | 0.16 | .4 | 0.85-1.47 |
| Participation reference value 0 = participants without valid 24-hour movement behavior data (n = 9 978), 1 = participants with valid 24-hour movement behavior data (n = 8 114).  OR = odds ratio; SE = standard error; CI = confidence interval. | | | | |

**Supplementary Figure S1**. Distribution of inverse probability weights

**
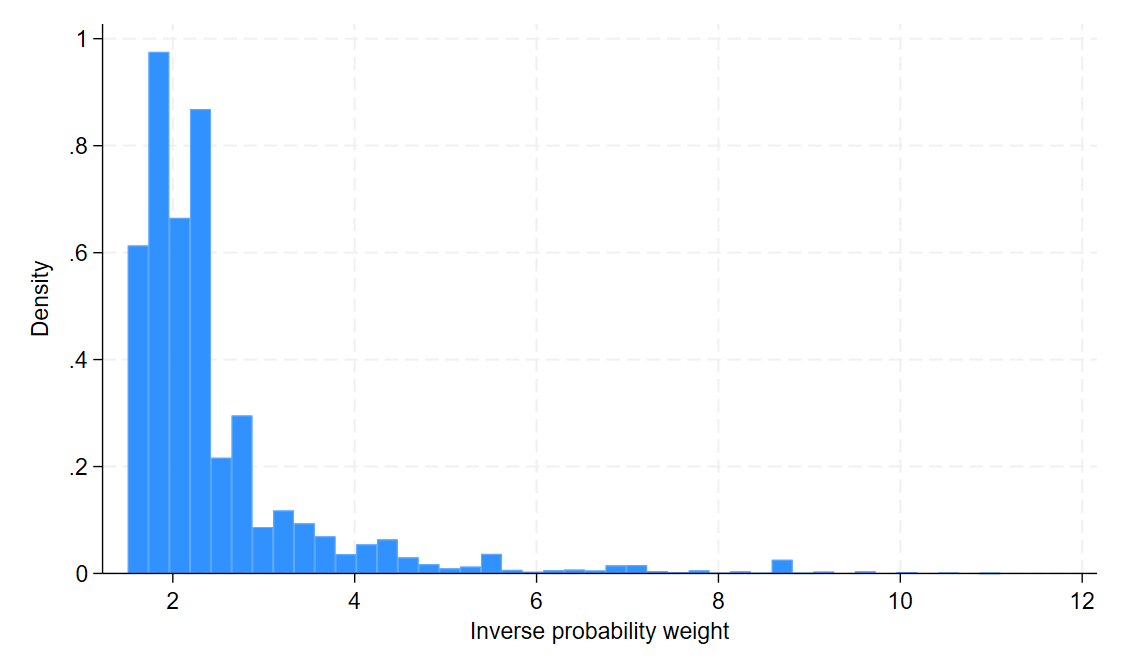
**

The distribution of inverse probability weights (IPW) used in the main analysis. The X-axis represents the IPW values, and the Y-axis indicates the density of each weight range in the sample. Weights are primarily centered around 1–2, indicating moderate inclusion probabilities. The right-skewed tail suggests a few individuals with relatively low inclusion probabilities, resulting in larger weights.

**Supplementary Table S3.** Crude estimates of background characteristics and movement behavior (min/24-hour) for all study participants and by sex

|  | **Total** | **Women** | **Men** |
| --- | --- | --- | --- |
|  | **(N = 8,114)** | **(n = 4,266)** | **(n = 3,848)** |
| Age, years, mean (SD) | 73.5 (6.3) | 73.7 (6.4) | 73.3 (6.1) |
| Education, years, n (%) |  |  |  |
| 0–10 | 1,519 (18.7) | 920 (21.6) | 599 (15.6) |
| 11–13 | 4,486 (55.3) | 2,276 (53.3) | 2,210 (57.4) |
| ≥14 | 2,109 (26.0) | 1,070 (25.1) | 1,039 (27.0) |
| Marital status, n (%) |  |  |  |
| Unmarried | 382 (4.7) | 160 (3.8) | 222 (5.8) |
| Divorced | 948 (11.7) | 544 (12.7) | 404 (10.5) |
| Widow/ -er | 1,313 (16.2) | 1,014 (23.8) | 299 (7.7) |
| Married | 5,471 (67.4) | 2,548 (59.7) | 2,923 (76.0) |
| Self-rated health, n (%) |  |  |  |
| Poor | 96 (1.2) | 50 (1.2) | 46 (1.2) |
| Not so good | 2,139 (27.1) | 1,207 (29.2) | 932 (24.8) |
| Good | 4,895 (62.1) | 2,486 (60.2) | 2,409 (64.1) |
| Very good | 754 (9.6) | 385 (9.3) | 369 (9.8) |
| BMI, kg/m^2^, mean (SD) | 27.1 (4.2) | 26.9 (4.5) | 27.4 (3.8) |
| Underweight | 51 (0.6) | 49 (1.1) | 2 (0.1) |
| Normal weight | 2,387 (29.6) | 1,443 (34.0) | 944 (24.7) |
| Overweight | 3,704 (45.9) | 1,707 (40.2) | 1,997 (52.3) |
| Obesity | 1,928 (23.9) | 1,051 (24.7) | 877 (23.0) |
| Wear time accelerometers |  |  |  |
| 1–7 days, mean (SD) | 5.8 (0.9) | 5.8 (0.9) | 5.8 (0.8) |
| 1–2 days, n (%) | 193 (2.4) | 114 (2.7) | 79 (2.0) |
| ≥3 days, n (%) | 7,921 (97.6) | 4,125 (97.3) | 3,769 (98.0) |
| Movement behavior, min/24-h, mean (SD) |  |  |  |
| Standing | 252.1 (82.5) | 269.4 (82.6) | 233.0 (78.0) |
| Walking | 87.8 (38.8) | 85.8 (38.0) | 90.0 (39.6) |
| Running | 0.3 (1.9) | 0.1 (0.9) | 0.4 (2.5) |
| Cycling | 4.8 (7.4) | 3.7 (5.7) | 6.0 (8.9) |
| Sitting | 546.2 (112.2) | 534.6 (108.4) | 559.1 (115.0) |
| Lying (awake) | 123.8 (81.7) | 114.9 (74.8) | 133.6 (87.7) |
| Sleep | 424.3 (48.8) | 430.7 (47.4) | 417.1 (49.2) |
| BMI = body mass index; kg = kilograms; m = meters. Underweight <18.5; Normal weight = 18.5–24.9; Overweight = 25–29.9; Obesity ≥30. | | | |

**Supplementary Table S4**. Crude estimates of background characteristics and movement behavior (min/24-hour) by sex and age group

| **Age group** | **65–69** |  | **70–74** |  | **75–79** |  | **80­–84** |  | **85­–90** |  | **≥90** |  |
| --- | --- | --- | --- | --- | --- | --- | --- | --- | --- | --- | --- | --- |
| **Women** | ***n* = 1 429** |  | ***n* = 1 300** |  | ***n* = 837** |  | ***n* = 423** |  | ***n* = 184** |  | ***n* = 93** |  |
| Education, years |  |  |  |  |  |  |  |  |  |  |  |  |
| 0–10 | 193 (13.5) |  | 256 (19.7) |  | 196 (23.4) |  | 141 (33.3) |  | 83 (45.1) |  | 51 (54.8) |  |
| 11–13 | 792 (55.4) |  | 700 (53.8) |  | 465 (55.6) |  | 207 (49.0) |  | 74 (40.2) |  | 38 (40.9) |  |
| ≥ 14 | 444 (31.1) |  | 344 (26.5) |  | 176 (21.0) |  | 75 (17.7) |  | 27 (14.7) |  | 4 (4.3) |  |
| Married, | 980 (68.6) |  | 865 (66.5) |  | 485 (58,0) |  | 172 (40.7) |  | 42 (22.8) |  | 4 (4.3) |  |
| Self-rated health |  |  |  |  |  |  |  |  |  |  |  |  |
| Good/ Very good | 1,072 (76.4) |  | 915 (72.1) |  | 557 (68.8) |  | 220 (54.5) |  | 82 (49.1) |  | 25 (33.3) |  |
| BMI, kg/m^2^ | 27.1 (4.5) |  | 26.9 (4.6) |  | 26.8 (4.4) |  | 26.6 (4.7) |  | 26.1 (4.1) |  | 24.9 (3.7) |  |
| Movement behavior,  min/24-hour | |  |  |  |  |  |  |  |  |  |  |  |
| Standing | 279.2 (77.9) |  | 272.0 (79.6) |  | 265.8 (80.3) |  | 257.6 (83.9) |  | 255.6 (107.2) |  | 195.8 (103.0) |  |
| Walking | 99.0 (34.6) |  | 91.3 (35.9) |  | 80.3 (34.4) |  | 65.0 (32.8) |  | 48.5 (31.0) |  | 24.3 (24.0) |  |
| Running | 0.2 (1.2) |  | 0.1 (0.9) |  | 0.0 (0.2) |  | 0.0 (0.0) |  | 0.0 (0.0) |  | 0.0 (0.0) |  |
| Cycling | 4.2 (6.0) |  | 4.1 (6.1) |  | 3.3 (5.0) |  | 3.2 (5.8) |  | 2.0 (2.2) |  | 1.8 (2.2) |  |
| Sitting | 520.1 (102.4) |  | 528.4 (106.0) |  | 542.8 (106.1) |  | 556.9 (111.0) |  | 569.5 (127.3) |  | 596.4 (137.3) |  |
| Lying (awake) | 109.7 (70.8) |  | 111.4 (69.1) |  | 112.9 (69.9) |  | 123.3 (82.6) |  | 133.9 (87.8) |  | 185.1 (132.3) |  |
| Sleep | 426.7 (47.7) |  | 432.1 (43.9) |  | 434.0 (45.2) |  | 434.2 (49.4) |  | 427.6 (58.6) |  | 435.1 (68.6) |  |
| **Age group** | **65–69** | ***p*** | **70–74** | ***p*** | **75–79** | ***p*** | **80­–84** | ***p*** | **85­–90** | ***p*** | **≥90** | ***p*** |
| **Men** | ***n* = 1 347** |  | ***n* = 1 213** |  | ***n* = 720** |  | ***n* = 365** |  | ***n* = 151** |  | ***n* = 52** |  |
| Education, years |  | .07 |  | .02 |  | .01 |  | <.001 |  | .02 |  | .003 |
| 0–10 | 154 (11.4) |  | 187 (15.4) |  | 131 (18.2) |  | 67 (18.4) |  | 45 (29.8) |  | 15 (28.9) |  |
| 11–13 | 801 (59.5) |  | 690 (56.9) |  | 400 (55.6) |  | 212 (58.1) |  | 78 (51.7) |  | 29 (55.8) |  |
| ≥14 | 329 (29.1) |  | 336 (27.7) |  | 189 (26.2) |  | 86 (26.5) |  | 28 (18.5) |  | 8 (15.4) |  |
| Married | 1,018 (75.6) | <.001 | 949 (78.2) | <.001 | 553 (76.8) | <.001 | 267 (73.2) | <.001 | 104 (68.9) | <.001 | 32 (61.5) | <.001 |
| Self-rated health |  | .03 |  | .02 |  | .07 |  | .003 |  | .08 |  | .05 |
| Good/ Very good | 1,066 (79.8) |  | 911 (76.4) |  | 485 (69.7) |  | 228 (65.1) |  | 65 (47.4) |  | 23 (51.1) |  |
| BMI, kg/m^2^ | 27.8 (3.8) | <.001 | 27.7 (3.7) | <.001 | 27.1 (3.7) | .2 | 26.7 (3.79 | .8 | 26.2 (3.3) | .9 | 25.5 (3.2) | .5 |
| Movement behavior,  min/24-hour | |  |  |  |  |  |  |  |  |  |  |  |
| Standing | 243.6 (75.3) | <.001 | 235.4 (77.4) | <.001 | 227.0 (76.1) | <.001 | 216.6 (76.1) | <.001 | 202.3 (88.8) | * | 192.2 (106.4) | .8 |
| Walking | 101.3 (37.6) | .09 | 94.6 (39.6) | .03 | 82.5 (35.1) | .2 | 70.8 (32.9) | .01 | 52.3 (31.6) | .3 | 35.0 (30.3) | .02 |
| Running | 0.6 (3.1) | <.001 | 0.4 (2.6) | <.001 | 0.3 (2.0) | <.001 | 0.1 (0.7) | .01 | 0.0 (0.0) | .07 | 0.0 (0.0) | .2 |
| Cycling | 6.5 (9.1) | <.001 | 6.2 (9.2) | <.001 | 5.9 (8.9) | <.001 | 5.0 (8.5) | <.001 | 3.6 (4.4) | * | 2.5 (3.6) | .1 |
| Sitting | 539.6 (112.9) | <.001 | 557.6 (115.9) | <.001 | 574.7 (108.3) | <.001 | 581.5 (113.2) | .002 | 599.8 (124.6) | .03 | 607.1 (125.4) | .6 |
| Lying (awake) | 134.5 (86.5) | <.001 | 130.4 (88.7) | <.001 | 128.2 (78.3) | <.001 | 141.2 (90.4) | .003 | 153.2 (110.8.) | .7 | 150.0 (111.3) | .1 |
| Sleep | 413.5 (47.3) | <.001 | 415.1 (47.9) | <.001 | 419.4 (51.1) | <.001 | 422.9 (52.9) | .002 | 428.1 (52.2) | .9 | 453.3 (45.1) | .08 |
| Continuous variables are shown as means *(SD)* and categorical variables as counts (n) with percentages (%).  BMI = body mass index; kg = kilograms; m = meters.  *p* Difference between women and men within each age group; Chi^2^ for categorical variables (n, %), ttest for continuous variables (mean, SD) | | | | | | | | | | | | |

**Supplementary Table S5**. Weighted background characteristics by sex and age group

| **Age group** | **65–69** |  | **70–74** |  | **75–79** |  | **80­–84** |  | **85­–90** |  | **≥90** |  |
| --- | --- | --- | --- | --- | --- | --- | --- | --- | --- | --- | --- | --- |
| **Women** | **n = 1 429** |  | **n = 1 300** |  | **n = 837** |  | **n = 423** |  | **n = 184** |  | **n = 93** |  |
| Education, years |  |  |  |  |  |  |  |  |  |  |  |  |
| 0–10 | 193 (16.4) |  | 256 (23.1) |  | 196 (27.6) |  | 141 (38.1) |  | 83 (51.6) |  | 51 (58.5) |  |
| 11–13 | 792 (56.7) |  | 700 (54.1) |  | 465 (54.9) |  | 207 (47.8) |  | 74 (37.1) |  | 38 (38.5) |  |
| ≥ 14 | 444 (26.9) |  | 344 (22.5) |  | 176 (17.5) |  | 75 (14.1) |  | 27 (11.3) |  | 4 (3.0) |  |
| Married | 980 (67.1) |  | 865 (64.8) |  | 485 (55.7) |  | 172 (38.2) |  | 42 (21.4) |  | 4 (3.4) |  |
| Self-rated health |  |  |  |  |  |  |  |  |  |  |  |  |
| Good/ Very good | 1072 (72.3) |  | 915 (67.3) |  | 557 (63.4) |  | 220 (48.5) |  | 82 (42.2) |  | 25 (26.2) |  |
| BMI, kg/m^2^ | 27.2 (4.8) |  | 27.0 (4.8) |  | 26.9 (4.4) |  | 26.7 (4.3) |  | 26.1 (3.2) |  | 25.0 (2.6) |  |
| **Age group** | **65–69** | ***p*** | **70–74** | ***p*** | **75–79** | ***p*** | **80­–84** | ***p*** | **85­–90** | ***p*** | **≥90** | ***p*** |
| **Men** | **n = 1 347** |  | **n = 1 213** |  | **n = 720** |  | **n = 365** |  | **n = 151** |  | **n = 52** |  |
| Education, years |  | .09 |  | .05 |  | .003 |  | <.001 |  | .009 |  | .02 |
| 0–10 | 154 (14.2) |  | 187 (18.6) |  | 131 (22.0) |  | 67 (22.1) |  | 45 (35.9) |  | 15 (38.7) |  |
| 11–13 | 801 (60.4) |  | 690 (58.0) |  | 400 (55.8) |  | 212 (58.4) |  | 78 (49.1) |  | 29 (51.6) |  |
| ≥14 | 329 (25.4) |  | 336 (23.4) |  | 189 (22.2) |  | 86 (19.5) |  | 28 (15.0) |  | 8 (9.7) |  |
| Married | 1018 (73.9) | <.001 | 949 (76.6) | <.001 | 553 (74.9) | <.001 | 267 (71.0) | <.001 | 104 (64.5) | <.001 | 32 (56.5) | <.001 |
| Self-rated health |  | .07 |  | .03 |  | .6 |  | .003 |  | .06 |  | .09 |
| Good/ Very good | 1066 (75.6) |  | 911 (71.5) |  | 485 (64.8) |  | 228 (59.8) |  | 65 (39.2) |  | 23 (41.0) |  |
| BMI, kg/m^2^ | 27.9 (4.0) | <.001 | 27.8 (3.8) | <.001 | 27.1 (3.9) | .3 | 26.8 (3.5) | .8 | 26.2 (2.6) | .8 | 25.5 (2.2) | .6 |
| Continuous variables are shown as weighted means (SD) and categorical variables as unweighted counts (n) with weighted percentages (%).  BMI = body mass index; kg = kilograms; m = meters.  *p* Differences between women and men within each age group, survey-weighted ordinal logistic regression for education; -binary logistic regression for marital status and self-rated health; and -linear regression for BMI. | | | | | | | | | | | | |

**Supplementary Table S6**. Weighted movement behavior (min/24-hour) by sex and age group

| **Age group** | **65–69 y** |  | **70–74 y** |  | **75–79 y** |  | **80­–84 y** |  | **85­–90 y** |  | ≥**90 y** |  |
| --- | --- | --- | --- | --- | --- | --- | --- | --- | --- | --- | --- | --- |
| **Women** | ***n* = 1 429** |  | ***n* = 1 300** |  | ***n* = 837** |  | ***n* = 423** |  | ***n* = 184** |  | ***n* = 93** |  |
| Standing | 278.0 (82.0) |  | 270.1 (84.2) |  | 264.5 (83.5) |  | 256.3 (78.7) |  | 250.7 (83.0) |  | 192.5 (64.3) |  |
| Walking | 97.7 (36.7) |  | 89.8 (37.8) |  | 78.6 (35.3) |  | 63.7 (30.4) |  | 45.6 (23.1) |  | 22.7 (14.6) |  |
| Running | 0.2 (1.2) |  | 0.1 (0.8) |  | 0.0 (0.2) |  | 0.0 (0.1) |  | 0.0 (0.0) |  | - |  |
| Cycling | 4.1 (6.3) |  | 4.0 (6.2) |  | 3.3 (5.1) |  | 3.1 (5.1) |  | 1.9 (1.7) |  | 1.9 (1.4) |  |
| Sitting | 521.9 (108.2) |  | 530.8 (112.9) |  | 544.9 (109.6) |  | 558.3 (105.2) |  | 575.7 (100.6) |  | 596.4 (86.1) |  |
| Lying (awake) | 110.9 (77.2) |  | 112.5 (73.5) |  | 113.6 (72.7) |  | 124.6 (80.0) |  | 136.5 (69.2) |  | 187.8 (81.6) |  |
| Sleeping | 426.4 (51.0) |  | 432.1 (46.5) |  | 434.2 (46.5) |  | 434.1 (45.7) |  | 426.5 (47.5) |  | 438.5 (41.9) |  |
| **Age group** | **65–69** | ***p*** | **70–74** | ***p*** | **75–79** | ***p*** | **80­–84** | ***p*** | **85­–90** | ***p*** | **≥90** | ***p*** |
| **Men** | ***n* = 1 347** |  | ***n* = 1 213** |  | ***n* = 720** |  | ***n* = 365** |  | ***n* = 151** |  | ***n* = 52** |  |
| Standing | 242.0 (79.3) | <.001 | 233.0 (81.6) | <.001 | 225.9 (79.0) | <.001 | 215.3 (73.9) | <.001 | 196.0 (71.7) | <.001 | 193.3 (79.1) | .9 |
| Walking | 100.2 (39.8) | .08 | 92.7 (41.7) | .06 | 81.2 (36.8) | .2 | 68.7 (30.6) | .04 | 47.8 (24.6) | .5 | 31.9 (19.9) | .06 |
| Running | 0.6 (2.9) | <.001 | 0.4 (2.7) | <.001 | 0.3 (2.2) | .002 | 0.1 (0.6) | .02 | 0.0 (0.0) | .09 | 0.0 (0.0) | .3 |
| Cycling | 6.5 (9.5) | <.001 | 6.1 (9.5) | <.001 | 5.8 (8.9) | <.001 | 4.9 (7.7) | <.001 | 3.3 (3.1) | <.001 | 2.3 (2.1) | .4 |
| Sitting | 541.1 (119.6) | <.001 | 560.6 (122.8) | <.001 | 575.8 (112.5) | <.001 | 581.3 (111.0) | .009 | 602.9 (101.4) | .08 | 595.0 (84.1) | .9 |
| Lying (awake) | 136.1 (92.0) | <.001 | 132.0 (93.2) | <.001 | 130.2 (82.7) | <.001 | 144.5 (88.9) | .004 | 162.1 (93.4) | .06 | 162.6 (77.3) | .3 |
| Sleeping | 413.2 (50.3) | <.001 | 414.8 (50.6) | <.001 | 418.8 (53.5) | <.001 | 423.7 (49.9) | .005 | 426.8 (43.5) | .9 | 455.4 (32.6) | .1 |
| Values are weighted means (*SD*), min/24-hour  *p* Differences between women and men within each age group were tested using survey-weighted linear regression | | | | | | | | | | | | |

**Supplementary Figure S2**. Predicted marginal effects of sex (men) across age on 24-hour movement behavior

**
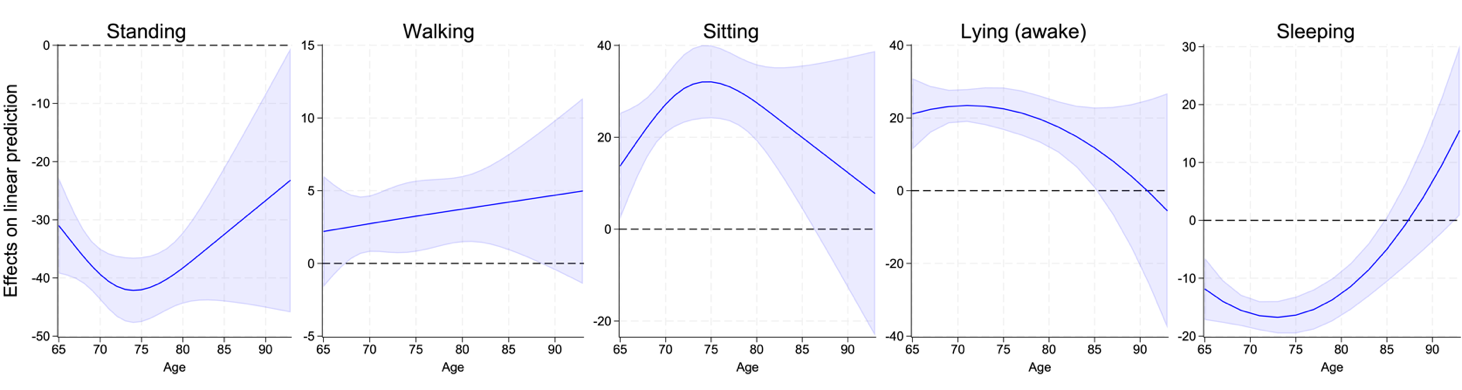
**

Predicted marginal effects of sex (men) across age on standing, walking, sitting, lying (awake), and sleeping, with 95% confidence intervals. Positive values indicate more predicted minutes for men than women. Negative values indicate fewer predicted minutes for men.

**Supplementary Figure S3**. Predicted marginal effects of education (<14 years) on 24-hour movement behavior


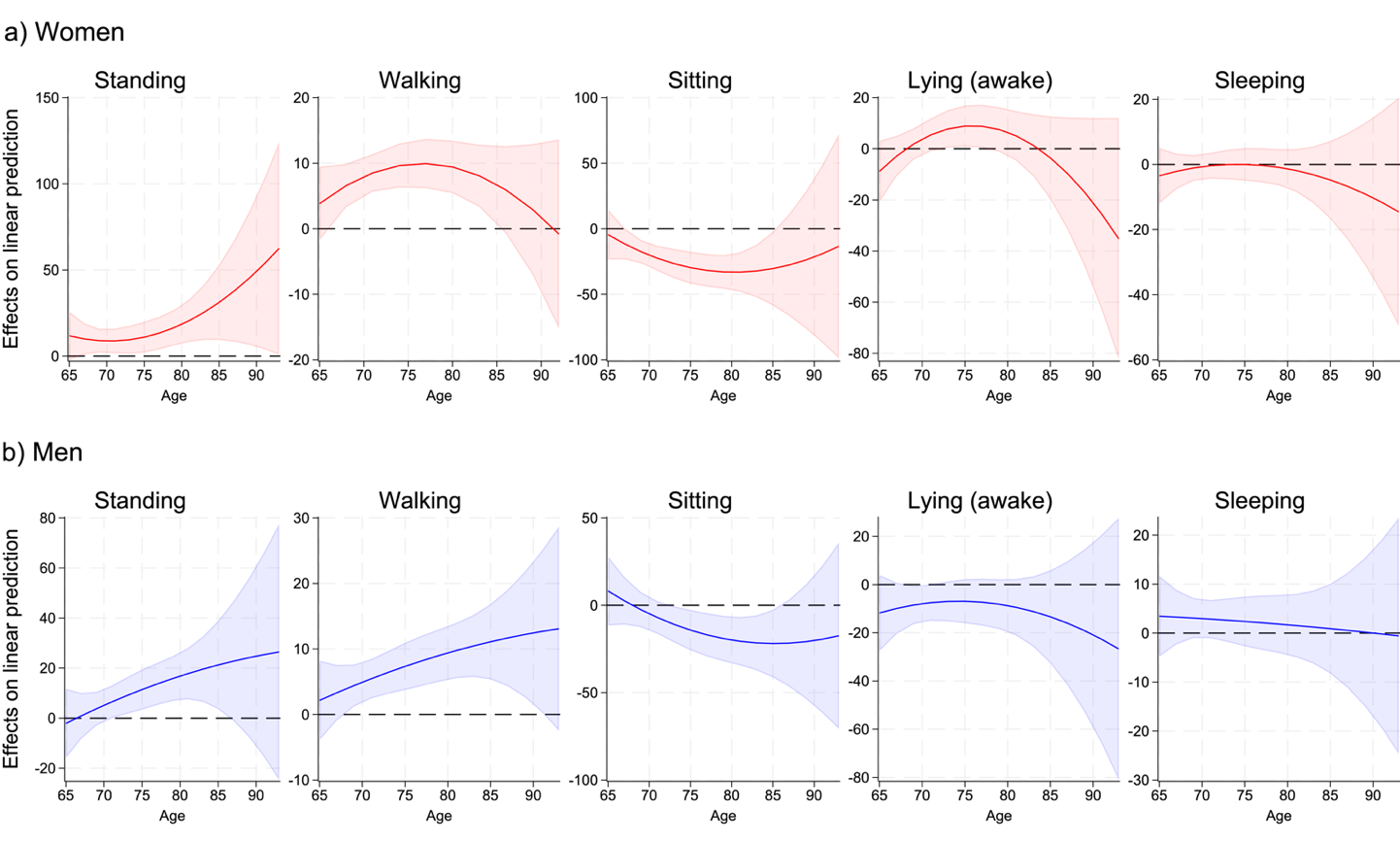


Predicted marginal effects of education (<14 years) on 24-hour movement behavior. a) Predicted marginal effects of education (<14 years), with 95% confidence intervals, on standing, walking, sitting, lying (awake), and sleeping for women across age. Positive values indicate fewer predicted minutes for women with <14 years education than those with ≥14 years education. Negative values indicate more predicted minutes for women with <14 years education than those with ≥14 years education. b) Predicted marginal effects of education (<14 years), with 95% confidence intervals, on standing, walking, sitting, lying (awake), and sleeping for men across age. Positive values indicate fewer predicted minutes for men with <14 years education than those with ≥14 years education. Negative values indicate more predicted minutes for men with <14 years education than those with ≥14 years education.
